# Selections

**Published:** 1894-08

**Authors:** 


### Selections.

### NEED OF INDEPENDENT MEDICAL JOURNALS.

There are two fundamental curses under which the profession in America labors:

1. The lack of independent medical journals controlled and issued by medical men for medical purposes. At present almost every journal is owned by some publishing or manufacturing concern, which of course publishes it for purely commercial purposes. It is easy to see that in such cases the advertising pages seek to control the reading columns. If an editor shows independence, and refuses to permit the commercial debauchery of his columns, woe be to him!

2. Co-operating with—nay, bound up with—this first misfortune, is the second,—the lack of a national or unitary organization. It at present seems to be impossible to arouse in the professional mind the recognition of the need of professional unity. Without professional unity we cannot speak to the people, criticise abuses, or encourage faint and sporadic attempts at virtue. We have no singleness or strength of voice, journalistic or organic; and from the multiplicity of non-co-operating jealous societies, together with the multiplicity of trade-journals, our professional disorder of speech is either a sorry laryngismus paralyticus or a sorrier laryngismus stridulus, whilst the omnipresent quack and patent-medicine men and the "likes of them" crowd every farm with nasty advertisements and huge bulletin-boards, and bribe to silence or a worse subserviency every secular newspaper of the continent.—*Philadelphia Letter in British Medical Journal.*

### IS LYSOL POISONOUS?

In answer to the question, Is lysol poisonous? which has been raised in consequence of a death in Bremen from the misuse of the article, Dr. Richard Drews, the children's physician in Hamburg, negatives the suggestion most decisively by an interesting case of a boy, only four years old, drinking undiluted lysol. The child, it was ascertained, had drunk about twenty-five grammes of lysol, but the nurse-girl in charge, out of fear, maintained silence in respect to it, until the child, which smelt strongly of the disinfectant, became after the mid-day meal suddenly very white, complained of pains in the head and limbs, and vomited violently. Up to that time he had continued to play as usual. When the doctor was called the child was slightly cyanotic, the pulse 56 and scarcely to be felt, whilst the respiration was superficial and sometimes in abeyance. The stomach was immediately washed out, removing a turbid, soapy liquid, smelling strongly of lysol, the odor of which still clung to breath and mouth after five washings.

After the stomach had been washed out the boy wanted something to drink, and was given milk, the pulse and respiration having meanwhile become normal and the cyanosis disappeared. The child also wanted to get out of bed, saying that it no longer had headache, but was kept in bed until the following morning with no other nourishment than milk. The next morning he exhibited not the slightest sign of indisposition, although the breath still smelt of lysol, the odor of which did not disappear until the third day. He said that the lysol had tasted nice, like beer. Evidently the twenty-five-gramme dose had only caused a slight temporary irritation of the nervous system. Dr. Drews is therefore of the opinion that one- to two-per-cent. lysol solutions can be employed in infantile practice without any fear of harm, and agrees with Dr. Landau, of Frankenburg, as to its freedom from poisonous qualities. . . .

In gynæcology and midwifery Dr. Rossa, of the Graz clinic, also emphasizes the extremely slight toxicity of lysol as compared with its high disinfectant value. For personal and external disinfection he recommends a one-per-cent. solution, and a two-per-cent. solution for gynæcological operations. The latter causes a violent burning of the mucous membranes, in one case local urticaria, and in another acute eczema. Compared with carbolic acid and corrosive sublimate in a number of births, lysol showed a larger percentage of feverless lyings-in, and a smaller number of cases with high fever. In the carbolic acid and corrosive sublimate period two fatal cases of puerperal sepsis occurred, in the lysol period none. Dr. Rossa also found lysol especially suitable in two-per-cent. solution for washing out the uterus in incipient endometritis puerperalis.—*Notes in New Remedies.*

